# Synergistic Effect of Carbon Micro/Nano-Fillers and Surface Patterning on the Superlubric Performance of 3D-Printed Structures

**DOI:** 10.3390/ma17051215

**Published:** 2024-03-06

**Authors:** Katerina Gkougkousi, Alexandros E. Karantzalis, Pantelis G. Nikolakopoulos, Konstantinos G. Dassios

**Affiliations:** 1 Department of Chemical Engineering, University of Patras, Caratheodory 1, 26504 Patras, Greece; gougousi.kat@gmail.com; 2 Department of Materials Science and Engineering, University of Ioannina, 45110 Ioannina, Greece; akarantz@uoi.gr; 3 Department of Mechanical Engineering and Aeronautics, University of Patras, 26504 Rio Patras, Greece; pnikolakop@upatras.gr

**Keywords:** superlubricity, friction, wear, 3D printing, graphene

## Abstract

Superlubricity, the tribological regime where the coefficient of friction between two sliding surfaces almost vanishes, is currently being investigated as a viable route towards the energy efficiency envisioned by major long-term strategies for a sustainable future. This current study provides new insights towards the development of self-lubricating systems by material and topological design, systems which tend to exhibit near-superlubric tribological performance, by reporting the synergistic effect of selective surface patterning and presence of carbon micro/nano-fillers on the frictional coefficients of additively manufactured structures. Geometric and biomimetic surface patterns were prepared by fused deposition modelling (FDM), using printing filaments of a polymeric matrix infused with graphene nanoplatelets (GNPs) and carbon fibers (C_f_). The calorimetric, spectroscopic, mechanical and optical microscopy characterization of the starting materials and as-printed structures provided fundamental insights for their tribological characterization under a ball-on-disk configuration. In geometrically patterned PLA-based structures, a graphene presence reduced the friction coefficient by ca. 8%, whereas PETG exhibited the lowest coefficients, in the vicinity of 0.1, indicating a high supelubric potential. Biomimetic patterns exhibited an inferior frictional response due to their topologically and tribologically anisotropy of the surfaces. Overall, a graphene presence in the starting materials demonstrated great potential for friction reduction, while PETG showed a tribological performance not only superior to PLA, but also compatible with superlubric performance. Methodological and technical challenges are discussed in the text.

## 1. Introduction

Material friction is the primary source of damage, wear and degradation in virtually any mechanical system involving movement. The phenomenon is responsible for the futile consumption of 20% of the world’s primary energy product [1]. Reducing friction not only saves significant amounts of energy but also considerably lowers the time and costs for maintenance of industrial equipment, which in turn, cuts down on plants’ downtimes and boosts production. It is predicted that a 20% reduction in friction in combustion engines can result in annual savings of EUR 120 billion and a 290-million-ton reduction in CO_2_ emissions [2]. Conventional friction-reducing agents are liquid lubricants which pose environmental concerns as, after the end of their lifespan, they need to be disposed of in landfills [3]. Research for the development of novel lubrication and anti-wear technologies, that will not contribute to waste and will minimize maintenance costs and times, is indispensable for supporting major future strategies such as the European Commission’s Green Deal roadmap, which prioritizes the environment and energy for a climate-neutral Europe by the year 2050 [4].

The concept of superlubricity, the tribological regime where the coefficient of friction decreases to the point that it almost vanishes, was first introduced by Hirano in 1990 [5]. The theoretical prediction suggests that when two crystalline surfaces come into incommensurate contact, the static friction between them becomes nearly negligible. This phenomenon, known as structural superlubricity (SSL), is observed during the sliding contact of two atomically smooth crystal surfaces in an incommensurate state [6]. It holds significant importance in the field of modern tribology, presenting a fresh perspective on lubrication [7]. This intriguing occurrence highlights the potential of utilizing two-dimensional (2D) layered materials not only as active components in nanoelectromechanical systems (NEMS), but also as effective lubricants for macroscopic applications. Early experiments have mainly focused on nanoscale interactions between layered materials, while only recently have the relevant experimental advancements expanded to the micron scale [8]. This progress towards structural superlubricity on a larger scale, represents a noteworthy stride towards practical implementation in future technologies.

Lee et al. pioneered the investigation of the frictional characteristics of two-dimensional (2D) materials such as graphene, MoS_2_ and h-BN, and indicated that, despite their thickness of only a few atomic layers, these materials exhibit friction-reducing actions comparable to those encountered in bulk lubricating agents [9]. In another study, Bustillos et al. reported that the embedment of such 2D materials in continuous phases as fillers for the production of composites significantly reduces the evidenced friction [10]. In that study, the friction coefficients reported for polylactic acid (PLA) matrix composites with embedded graphene nanoplatelets (GNPs) were 65% lower than those prepared from plain PLA, pointing towards superlubric behavior. The corresponding wear resistance of the composites was 14% higher than the reference value. Uzoma et al. [11] independently demonstrated the great potential of graphene in promoting the tribological behavior of surfaces by significantly reducing their friction coefficient.

Past studies have shown that friction is preferably suppressed on surfaces featuring some type of structural patterning, as compared to microscopically, or even atomically, flat ones [7,12,13,14]. Surface patterning is a highly popular strategy for the enhancement of the macroscale tribological performance of materials, due to the dual role of the pattern: under hydrodynamic lubrication, it improves the mechanical performance of the lubricating film, while under dry sliding, it acts as a wear debris depository. The observation was first made in nature, where friction was found to be significantly reduced in non-smooth surfaces such as shark skin [15], fish scales [16] and snake scales [17,18,19]. Geometric patterning for the same scope involves the deliberate introduction of convex or concave microgrooves, dimples and shape arrays on surfaces by techniques such as laser texturing, electrical discharge texturing, etching and oxidation [5]. Both biomimetic (inspired from nature) and geometrical surface micropatterning for friction reduction are gaining increased attention in tribological research [7].

As a fully customizable and diverse rapid prototyping technique, adaptable to highly complex shapes, three-dimensional (3D) printing offers a plausible manufacturing approach for patterned surfaces for friction and wear reduction across different-length scales. Three-dimensional printing technologies can be categorized into seven main categories, namely, (i) vat photopolymerization, which uses an energy source like a laser beam to selectively cure a layer of photosensitive materials (e.g., stereolithography, digital light processing (DLP)); (ii) material extrusion, where materials are dispensed through an extrusion nozzle (e.g., fused deposition modelling (FDM), direct ink writing); (iii) powder bed fusion, where a powder bed is fused and sintered via a laser or an electron beam (e.g., selective laser sintering, selective laser melting (SLM)); (iv) binder jetting, which uses inkjet technology to selectively deposit binder onto the powder bed; (v) material jetting, where liquid droplets are deposited on a substrate (e.g., inkjet printing (IJP)); (vi) sheet lamination, where the part is built by the lamination of sheet feedstock (e.g., laminated object manufacturing), and (vii) directed energy deposition, wherein powder or wire is combined with a laser to deposit materials onto a build plate [7]. Among the various 3D printing techniques, SLM, DLP, IJP and FDM are the most cited, with the latter being one of the most widely used 3D printing technologies. FDM involves feeding the starting filament into a nozzle, where it is melted and then deposited onto a heated horizontal surface, to form a pre-designed shape [20,21]. It has drawn tremendous tribological attention, and diverse lubrication structures have been manufactured by various additive manufacturing routes, as reviewed in [5]. PLA is probably the most common 3D printing filament, owing to its ease of use, recyclability, compostability and non-toxicity as it is made from corn starch, [22]. At the same time, polyethylene terephthalate glycol-modified (PETG) filaments are currently emerging as one of the most versatile and reliable alternatives, due to their perfect balance between strength and flexibility, their durability, chemical resistance, excellent bed adhesion, minimal warping, fairly low printing temperatures, moisture resistance, and odorless nature, with low fume emissions during the printing process.

The current study investigates and reports on the combined effect of surface micropatterning and carbon micro/nano-fillers on the tribological performance of biomimetic and geometrical structures produced by FDM from polymeric starting filaments enhanced with graphene nanoplatelets and short carbon fibers. While relevant recent literature reports a very wide range of structures, experimental protocols and starting materials, the present work offers the novelty of a systematical comparison between geometric and biomimetic patterns additively manufactured under identical experimental protocols from the same specialty filaments, with or without micro- and nano-fillers that can promote superior shear performance and self-lubrication in the absence of external lubricants. This enables the unique possibility of a direct assessment of the effects of filler presence, type and pattern type, bioinspired and geometric, on the tribological performance of structures. Critical experimental challenges are identified and discussed.

## 2. Materials and Methods

### 2.1. Materials

To study and compare the effect of carbon fillers of different length-scales on the frictional behavior of 3D printed structures, three types of printing filaments were used, namely pure PLA, composite PLA with embedded graphene nanoplatelets (commercial name GRAFYLON3D) and composite polyethylene terephthalate glycol-modified (PETG) with 10% wt. embedded carbon fibers (PETG-C_f_, commercial name ALFAOMNIA). All filaments were 1.75 mm in diameter and were purchased from FILOALFA Maip Compounding S.r.l. (Torino, Italy). As a neat PETG filament was not available from the PETG-C_f_ filament provider, a stock pure PETG filament from a different manufacturer (NEEMA3D S.A., Athens, Greece) was used to produce corresponding specimens for tensile testing and assessing the effect of carbon fibers on the polymer’s mechanical behavior.

### 2.2. Fused Deposition Modelling

**Figure 1:**
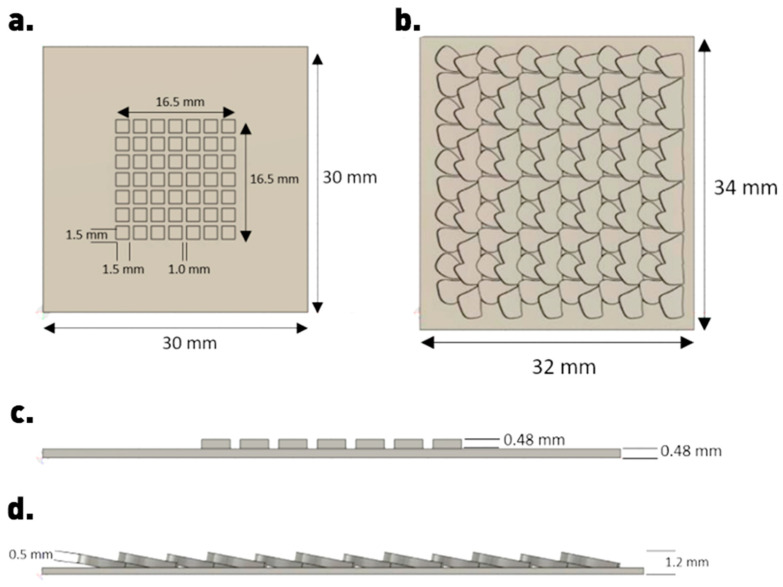
Dimensional characteristics of as-designed geometric cubic structures (**a**,**c**) and biomimetic snakeskin structures (**b**,**d**).

Structure design was performed in Autodesk Fusion 360 software (Education version, Autodesk Inc., San Francisco, CA, USA). The geometric pattern for tribological investigation was chosen as a cubic arrangement, based on previous findings of friction coefficient reduction in the case of copper nanoparticles combined with a liquid lubricant carrier [23]. For the biomimetic pattern, a snakeskin texture was chosen, inspired by the ease of movement of snakes in dry environments in the absence of any liquid carrier on their skin. The particular pattern was designed from scratch in the present study and has not been tested before. A previous simplified tribological study of three-dimensionally printed snakeskin structures omitted realistic considerations such as snake scale overlap, which is the case for the totality of the scale-covered body except the head [24]. Schematics of the as-designed geometric and biomimetic arrangements are depicted in Figure 1 along with their dimensional characteristics. The stereolithographic design file was imported into Ultimaker Cura 3D printing software (Version 4.0, Ultimaker BV, Utrecht, The Netherlands) and converted into G-code programming language used to control the movement of the 3D printer.

Sample production by FDM was performed on a Creality3D CR10-V2 printer (Shenzhen Creality 3D Technology Co., Ltd., Shenzhen, China) featuring a heated carborundum glass build plate offering minimization of warping and the easy detachment of 3D prints. Nozzle and build plate temperatures were adjustable depending on the filament type. The specific printing parts’ temperatures were defined following differential scanning calorimetry (DSC) analysis for each material; the nozzle temperature was set to 190 °C for PLA and PLA-graphene, 245 °C for PETG and PETG–carbon fibers, and bed temperatures were 60 °C and 100 °C for the two families, respectively.

### 2.3. Spectroscopy and Microscopy

The quality of carbon fillers in composite PETG-C_f_ filaments was assessed using Raman spectroscopy and optical microscopy. The primmer investigations were conducted on an inVia™ confocal Raman microscope (Renishaw PLC, Gloucestershire, UK) using a solid-state laser with a 514 nm wavelength as the excitation source. The ratio of intensities of the D and G peaks in carbon’s Raman spectra is representative of the degree of crystallinity and structural defects in the carbon, while it is can also be correlated to the number of graphenic layers in the corresponding nanoplatelets [23].

For the optical observation of the cross-sectional and longitudinal sections of composite filaments, small pieces of each were encapsulated in typical mounting resin disks by immersion in a resin/hardener mixture at a weight ratio of 35/100. After a hardening cycle of 48 h, the capsule surface was polished in a rotational grinding machine, initially with 500 and 1000 grade paper to bring the carbon fillers of the filaments to the surface, and then with 2000 and 4000 grades for the final polishing that eliminated surface imperfections and optimized the optical characterization. The same method was used to produce capsules with filament lengths placed both parallel and perpendicular to the examination surface.

### 2.4. Mechanical Testing

**Figure 2:**
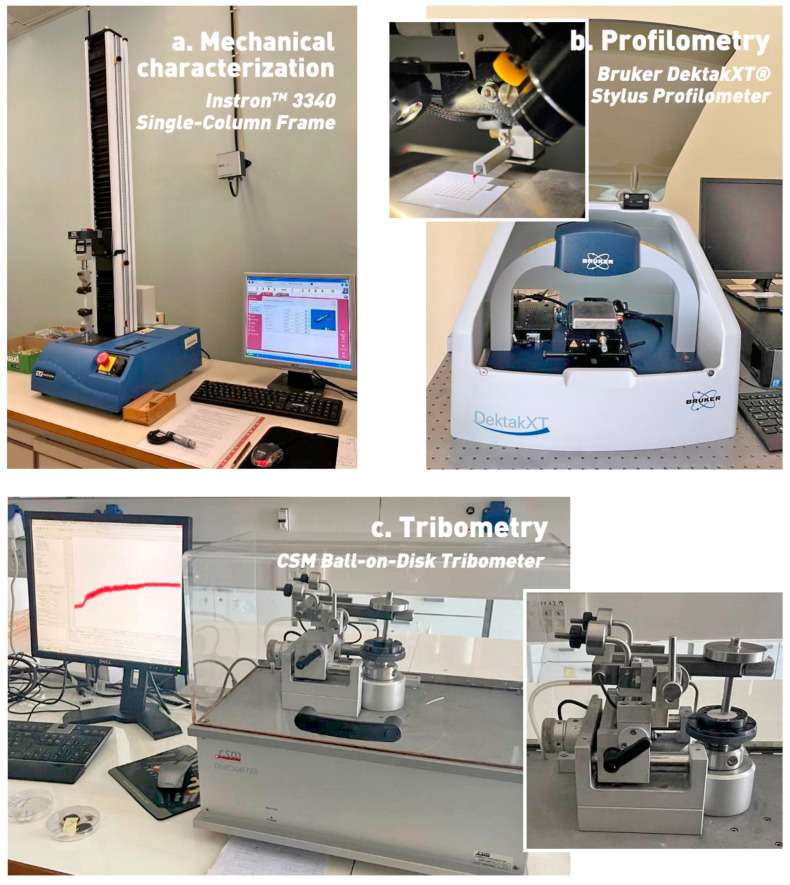
Main instrumentation used for (**a**) mechanical testing, (**b**) surface topography and roughness and (**c**) tribometry.

The mechanical behavior of the developed materials was characterized under monoaxial tension in an Instron^®^ 3340 mechanical testing frame (Instron Corp., Norwood, MA, USA), Figure 2a. The main purpose of the experiments was the establishment of the yield strengths of the as-printed materials, which would serve as input for the normal force range of the tribometry experiments for the determination of the surfaces’ friction coefficients under superlubricity-enabling conditions.

Five typical tensile test specimens were prepared and tested under uniaxial displacement-controlled tension, with a cross-head speed of 5 mm/s, for each polymeric filament type according to ASTM D-638-02 standard [25] test method specifications. In order to identically replicate the synthesis conditions, the tensile coupons were 3D printed themselves, following the same cycle used for the tribological structures. In fact, any other specimen fabrication method would not be representative of additive manufacturing specifics such as the small voids that are inevitably introduced into the material during fused deposition of the polymeric-based precursor.

### 2.5. Profilometry

The surface topography and roughness of the as-printed geometrical and biomimetic patterns of the three filament types was assessed in a Bruker DektakXT^®^ stylus profilometer (Bruker Corp., Billerica, MA, USA), Figure 2b. All measurements were performed at room temperature with a stylus of a tip diameter of 2.5 μm.

### 2.6. Tribometry

A CSM ball-on-disk tribometer (CSM Instruments SA, Peuseux, Switzerland) was used for friction and wear testing, Figure 2c. Samples under investigation were placed on a round base inside the instrument’s main chamber. On top of the sample rested a lever, which carried the desired weight (vertical load) and had the counter body (steel ball) at its end. Frictional forces, resulting from the rotational sliding of the level’s end (counter body) on the surface under investigation, were quantified by recording the small deviations of the lever, by means of two linear differential transformation (LVDT) sensors. The friction coefficient was thereafter typically obtained as the ratio between the sensor signal (friction force) and the imposed vertical load. A 6 mm diameter steel ball was used as the counter body, and the vertical loading was chosen as 55 MPa based on mechanical testing indications for the achievement of local stress levels marginally greater than the yield stress of the materials. The speed of the counter body on the sample was set to a standard value of 16 mm/s, and the motion of the ball was rotational on the sample. Measurements were performed at room temperature conditions (20 °C, 70% humidity) for a total duration of 3600 s (60 min) each.

## 3. Results and Discussion

### 3.1. Spectroscopy

**Figure 3:**
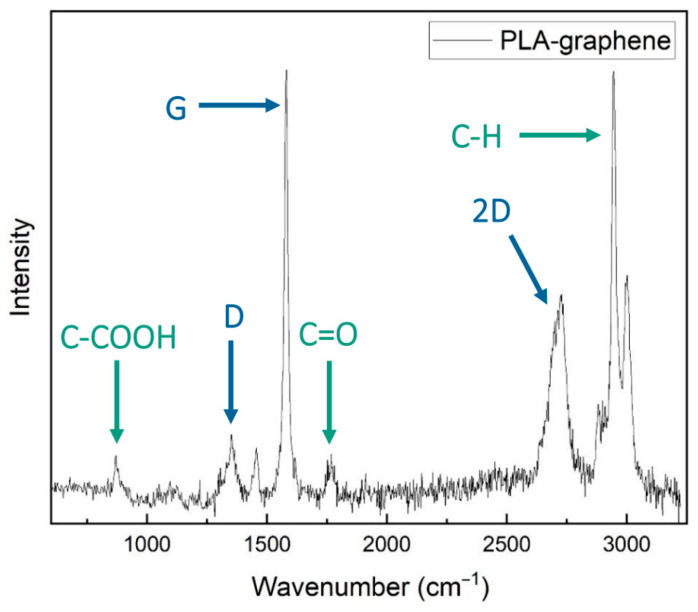
Raman spectra of composite PLA filaments with embedded graphene nanoplates.

The typical Raman spectroscopic response of the composite PLA–graphene filament is presented in Figure 3. Therein, the D, G and 2D carbon peaks are observed at wavelengths of 1353.5 cm^−1^, 1579.87 cm^−1^ and 2697.96 cm^−1^, respectively, in harmony with expectations for graphene. The intensity ratio of the D and G peaks, *I_D_*/*I_G_*, has a value of 0.136, which indicates that graphene in the composite has good crystallinity, without significant level of structural defects; the ratio also suggests that the nanocarbon is present in the form of nanoplatelets, each containing 10–60 layers of graphene [23]. In the same spectrum, the prominent peak identified at a wavelength of 2946.38 cm^−1^, as well as the two smaller peaks at 867.77 cm^−1^ and 1767.66 cm^−1^, are attributed to distinct chemical groups of the PLA phase of the filaments. As shown in Figure 3, the peak at 867.77 cm^−1^ represents the carboxyl group (-COOH), the peak at 1767.66 cm^−1^ represents the carbonyl group and the peak at 2946.38 cm^−1^ the carbon–hydrogen bonds.

**Figure 4:**
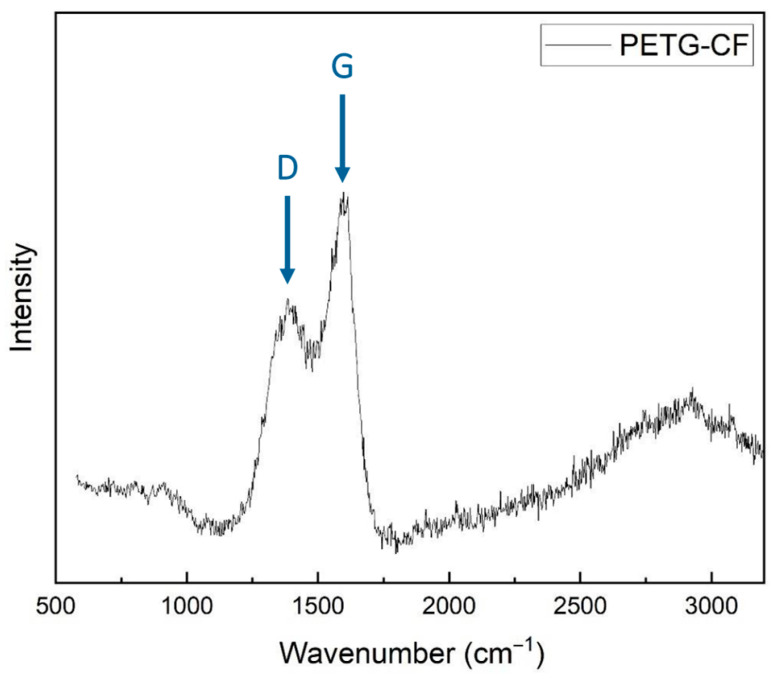
Raman spectra of composite PETG filaments with embedded carbon fibers.

Corresponding features were observed in the Raman spectra of PETG filaments with embedded carbon fibers, Figure 4. The peaks recorded at 1445.47 cm^−1^ and 1613.58 cm^−1^ correspond to the D and G peaks, respectively, of carbon from the fibers in the composite thread. PETG is expected to exhibit low-intensity peaks in the range of 1400–1600 cm^−1^, which are not distinguishable as convoluted into the higher-intensity peaks of carbon. Here, the *I_D_*/*I_G_* peak ratio value is 0.64.

### 3.2. Optical Microscopy

**Figure 5:**
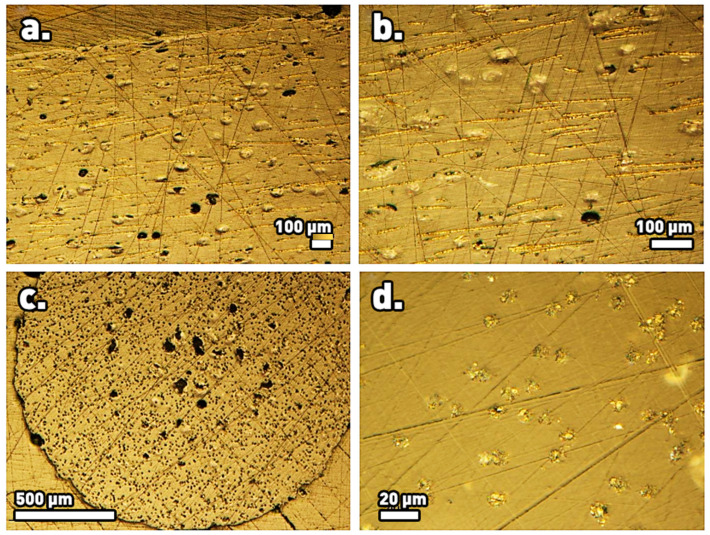
Optical microscopy images of interior of composite PETG filaments with embedded carbon fibers at different magnifications: (**a**,**b**) longitudinal sections and (**c**,**d**) cross sections.

**Figure 6:**
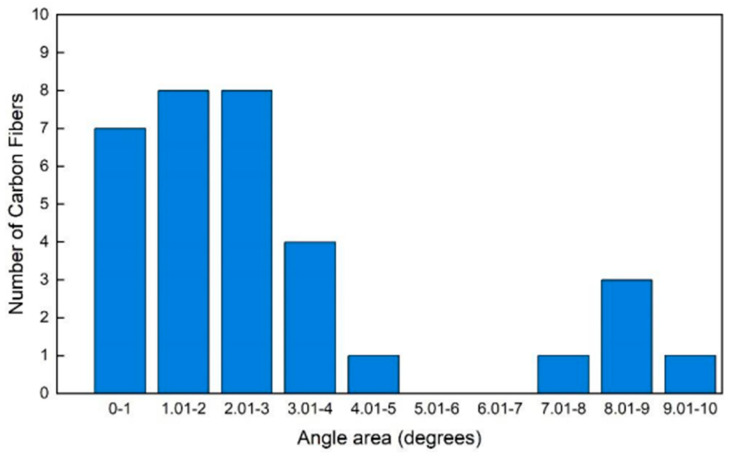
Statistical angle of misalignment, with respect to filament axis, of carbon fibers embedded in PETG composite filaments.

Optical microscopy imaging of the PETG filaments with embedded carbon fibers revealed that the fibers were mostly discontinuous and rested overall parallel both to the filament axis as well as to each other. These features are demonstrated in the micrographs of longitudinal sections of the filaments, Figure 5a,b. Cross-sectional views (Figure 5c,d) revealed undistorted circular cross-sections, which was also an indication of good overall fiber alignment (misaligned fibers would provide elliptical cross sections). The average angle of the fibers with respect to the macroscopic orientation of the thread axis was measured as 2.98 degrees, with 82% of the fibers remaining within a small misalignment angle of less than 4 degrees from the thread’s axis (Figure 6). The observed overall good alignment is typical of the extrusion method used for the production of the thread, wherein the mix of the polymeric matrix and carbon fibers is pushed through the die. It is worth recalling that the orientation of the fibers within the composite filament is crucial for its axial properties.

**Figure 7:**
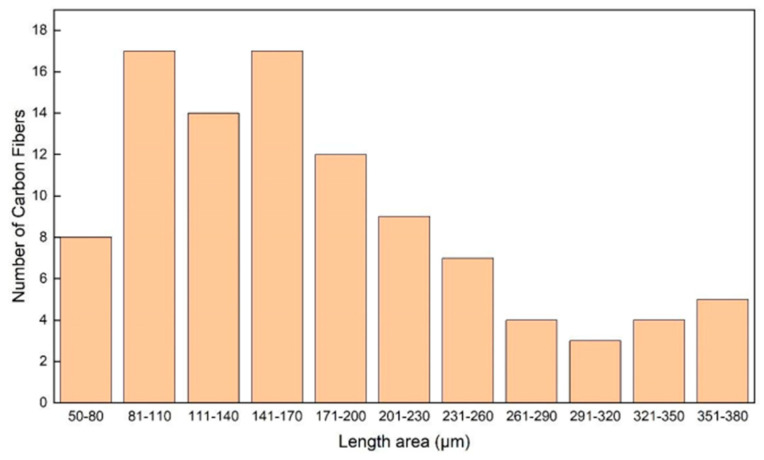
Range of carbon fiber lengths embedded in the PETG composite filaments.

An image analysis of the recorded micrographs performed in IC Measure software (Version 2.0.0.286, The Imaging Source Europe, Bremen, Germany) provided statistics of fiber length and diameter. Regarding the primmer (Figure 7), results from a population of 100 fibers showed that fiber length ranged from short values of 50 μm to quite long ones of up to 380 μm. The highest population was observed in the ranges of 81–110 μm (17%) and 141–170 μm (17%), followed by the intermediate range of 111–140 μm (14%) and that of 171–200 μm (12%). The remaining 40% of the fibers were distributed among the rest of the length ranges, with percentages ranging from 3% to 9% for each range. The average carbon fiber diameter was measured as 8.47 μm, a value which compares favorably with the expected range of diameters of typical carbon fibers used in composite materials—5–10 μm.

Optical microscopy observations of the internal structure of PLA filaments with embedded graphene nanoplatelets revealed longitudinal GNP-rich zones in the filaments, as expected from individual platelet feeds into the extrusion process, and an average platelet lateral dimension of 6.30 μm.

**Figure 8:**
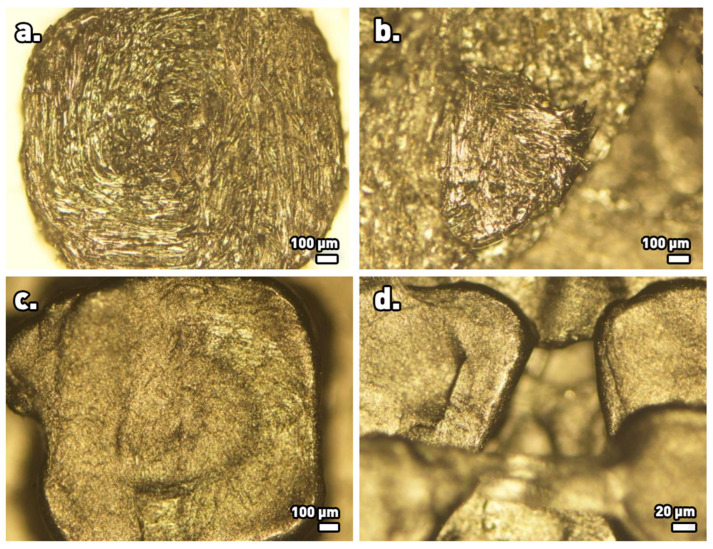
Optical microscopy images of 3D-printed structures at different magnification levels. Top row (**a**,**b**): structures made from composite PETG-C_f_ filaments. Bottom row (**c**,**d**): structures made from composite PLA-GNP filaments.

Observation of the surfaces of the final as-printed structures by optical microscopy revealed uniform distributions of the reinforcing carbon fibers and GNPs, without occurrence of apparent voids or agglomerations (Figure 8). In combination with the fact that the carbon filler distributions were not uniform in the originating threads, this observation leads to the conclusion that the fusion process of the filament during additive deposition contributes to filler homogenization in the resulting 3D-printed structure.

### 3.3. Profilometry Results

The profilometry results were indicative of a good quality of additive manufacturing, as the measured mean surface roughness of 37 μm (range of 25–50 μm) was of the same order of magnitude for both geometric and biomimetic structures, as well as among all filaments. Small discrepancies in the recorded surface roughness fell within the range of printing error, ±120 μm, and could be partially attributed to the different viscosities of the fused filament materials, especially in view of the differences in printing temperatures between materials. The printing error, or resolution of 120 μm, is a key factor in the accuracy of as-deposited structures; it was herein established that the printer behaved almost independently of filament type, and each surface is expected to have some micrometer-scale relief.

### 3.4. Mechanical Testing Results

**Figure 9:**
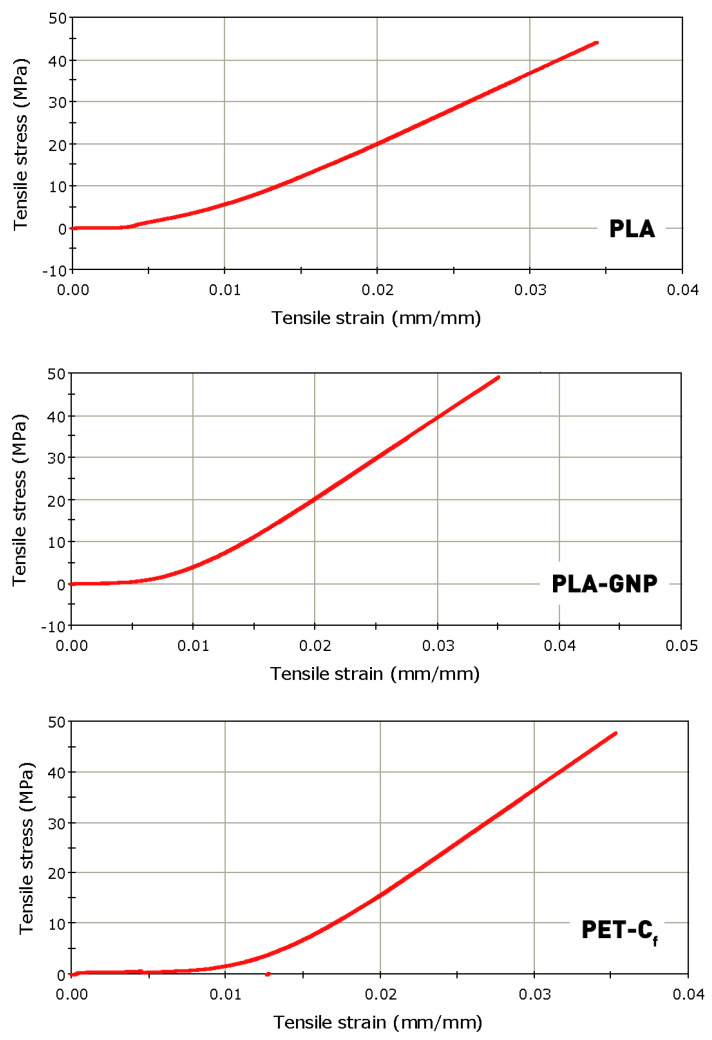
Typical stress–strain curves of tensile coupons, 3D printed following the same cycle as the tribological structures, for characterization of the mechanical properties of the as-printed materials.

The stress–strain responses of the materials provided their main mechanical properties, namely, the Young’s modulus, tensile strength and yield strength, as summarized in Table 1. Typical mechanical curves are depicted in Figure 9. The results indicate that the addition of graphene results in a 7.5% increase in the elastic modulus of PLA, while the addition of carbon fibers leads to a corresponding 59.6% increase in the modulus of PETG. It should be recalled that in the case of PETG, the neat and carbon-enhanced PETGs originate from different manufacturers and therefore may have undergone different thermal processing cycles or may contain different additives, which may rationalize some small discrepancies in the mechanical properties. Nonetheless, the observed improvement of ca. 60% cannot be attributed to such processing micro-differences and is believed to be the main effect of carbon fiber presence in the PETG-C_f_ composite filament.

Establishment of the yield strength of the materials was of key significance in the current study, as this value is desired as a minimum contact pressure limit during tribological measurements. In essence, the samples need to marginally yield to allow the manifestation of superlubricity. Based on the above results, a contact pressure of 55 MPa was chosen for the tribological measurements, common to all materials.

### 3.5. Tribology

**Figure 10:**
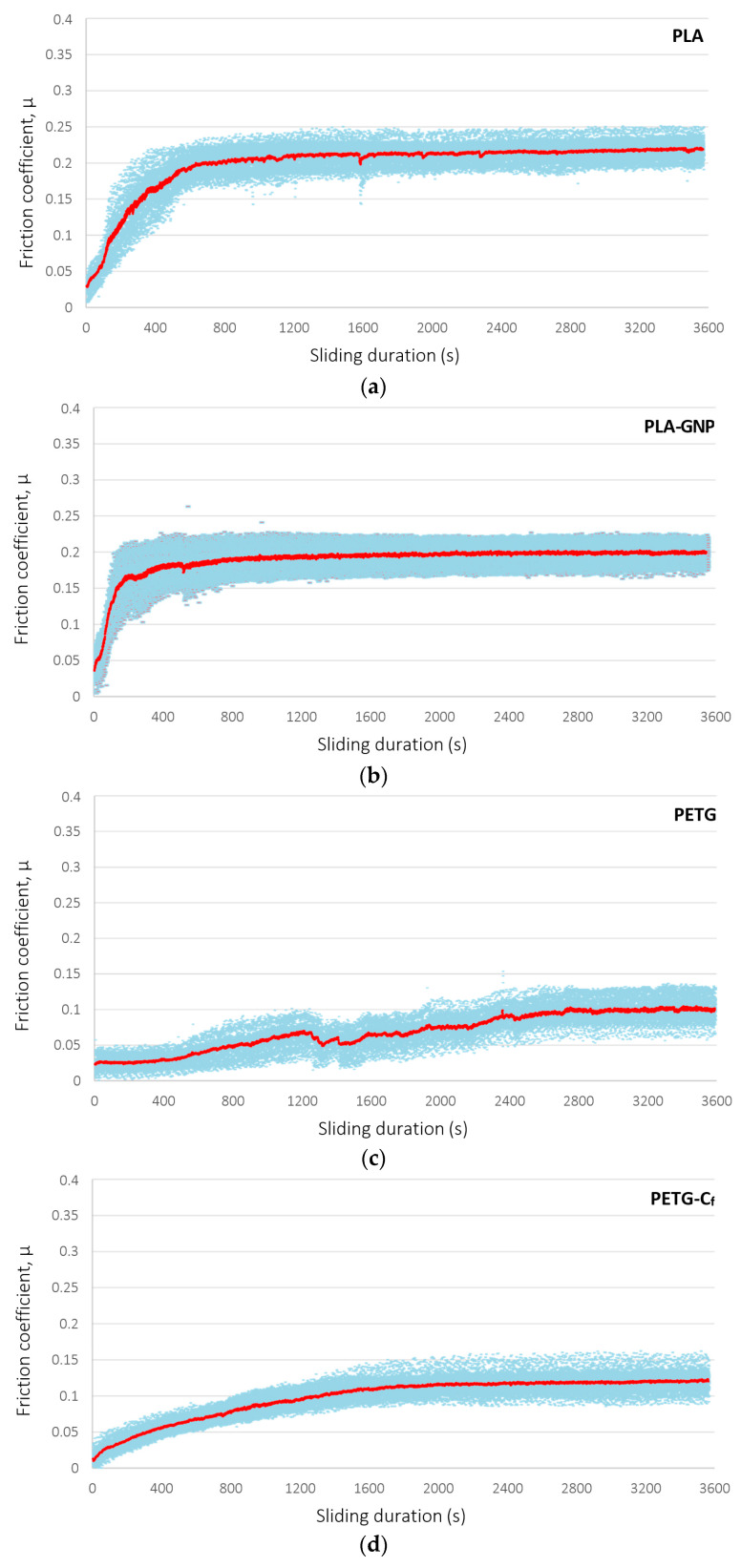
Temporal variation of friction coefficient, under ball-on-disk configuration, for geometric structures additively manufactured from four different precursors: (**a**) PLA, (**b**) PLA-GNP, (**c**) PETG, (**d**) PETG-C_f_. Blue symbols are the friction coefficient and red lines represent their mean values in each instance of the experiment.

The evolution of the friction coefficient during ball-on-disk tribological measurements of the geometric patterns (3D cube structure) is presented in blue symbols for the four materials under investigation, namely, plain neat PLA, PLA–graphene, PETG and PETG-C_f_, in Figure 10. In the diagrams, the red curves represent the mean value of the measurements, at each instance of the experiment. It is observed that a graphene presence in PLA reduces the coefficient of friction of the polymer by 7.8%. PETG has an overall significantly lower coefficient of friction, which increases by 21.8% with the addition of carbon fibers. The primmer observation, i.e., of a lower coefficient of friction of PETG compared to PLA, is consistent with the literature’s findings [26] and can be attributed to the amorphous structure of PETG, as opposed to the well-organized crystalline chains of the structure of PLA. This allows PETG to undergo greater deformations and experience smaller frictional forces when in contact with another surface, which gives rise to lower friction. In fact, PETG-related friction coefficients in the vicinity of 0.1 or less point to superlubric behavior. The second observation, i.e., of an increase in the friction coefficient due to the presence of carbon fibers, can be rationalized upon the random orientation of the high-aspect ratio carbon fibers in the as-printed sample, which follows the direction of printing and does not favor the tribological response of the material. The coefficients of friction are summarized for all structures and materials in Table 2.

**Figure 11:**
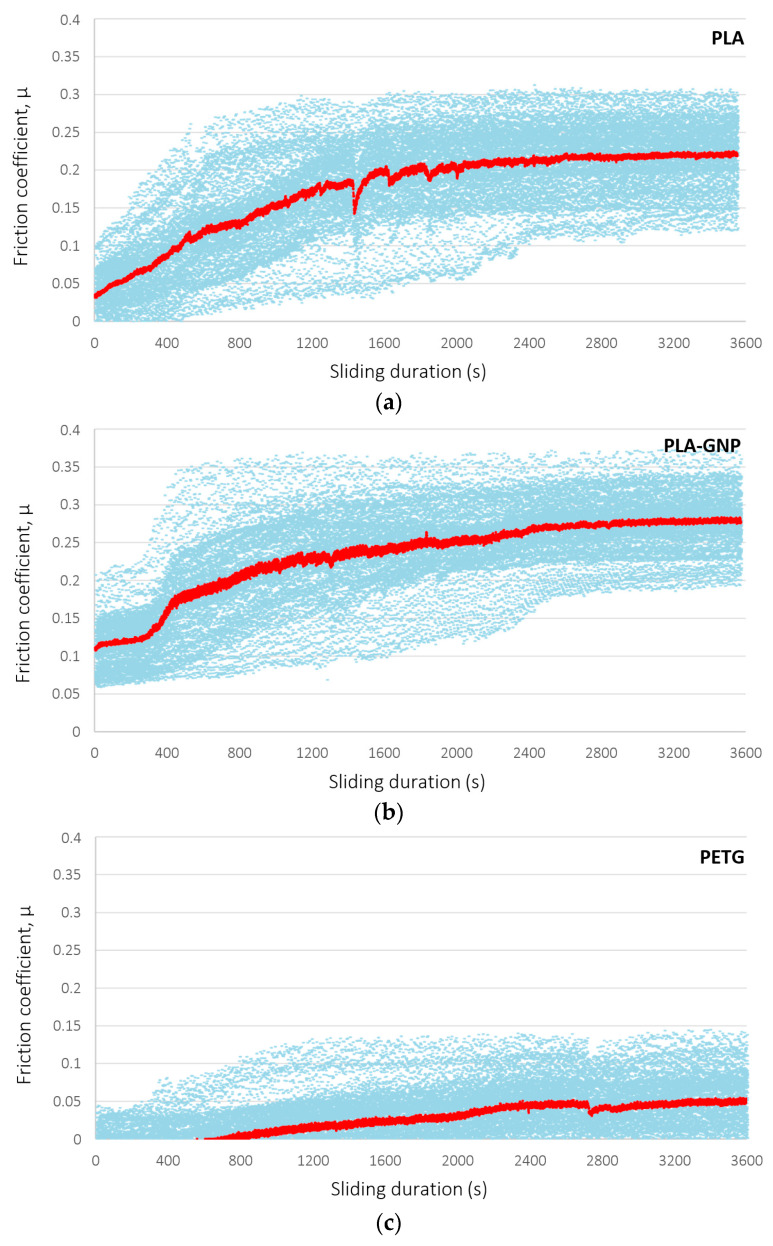

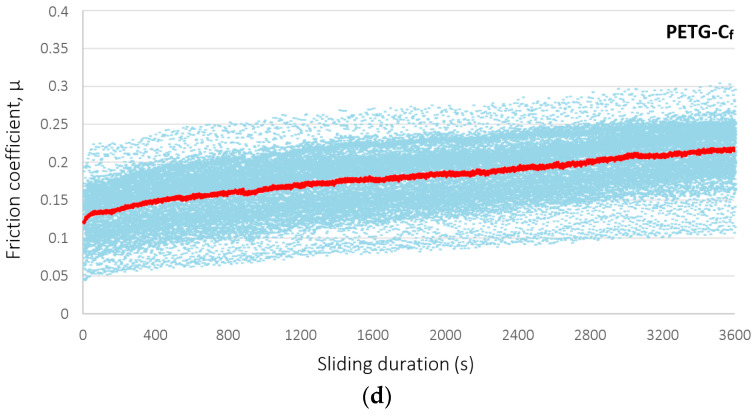
Temporal variation of friction coefficient, under ball-on-disk configuration, for biomimetic snakeskin structures additively manufactured from four different precursors: (**a**) PLA, (**b**) PLA-GNP, (**c**) PETG, (**d**) PETG-C_f_. Blue symbols are the friction coefficient and red lines represent their mean values in each instance of the experiment.

The corresponding tribological behavior of the biomimetic snakeskin patterns, 3D-printed by the same four filament materials, is presented in blue symbols in Figure 11. As previously, red curves in the diagrams represent mean values of the friction coefficient at each instance of the experiment. The considerable noise of the curves dominates the response and is attributed to the anisotropic nature of the surface. Snakeskin is structured to exhibit preferential tribological behavior only in the direction of movement of the serpent, whereas ball-on-disk tribology is performed by the circular movement of the counter body on the surface. This forces the counter body to partly slide along the intended direction of movement and partly strike the snake scales in the opposite direction, giving rise to noise. The same effect renders the contact area of the counter body with the sample surface irregular, which lowers the contact pressure and may inhibit material yield, which is indispensable for the manifestation of superlubricity. This effect may explain the 25.9% higher friction coefficient of PLA-GNP samples compared to plain PLA, as also observed in the values of Table 2. It is hence important that tribological measurements must follow any (an)isotropic features of the measured surfaces. Notwithstanding the fact that the measured friction coefficients of the biomimetic structures may significantly overestimate the real values along the sliding direction due to the above reasoning, it is concluded that the geometric (cube pattern) structure exhibits optimal tribological behavior compared to that of the snakeskin structure, with the PETG material, in particular, indicating superlubric behavior.

## 4. Conclusions

The synergistic effect of carbon-based micro/nano-fillers and surface micropatterning on the superlubric performance of additively manufactured structures from polymeric filaments, enhanced with graphene nanoplatelets and short carbon fibers, was investigated in the current study. Samples with geometrically and biomimetically patterned surfaces were produced by fused deposition modelling and tested tribologically under a ball-on-disk configuration with a steel counter body of 6mm diameter. The materials were initially characterized mechanically for the establishment of yield and tensile strengths and elastic modulus, which were found to vary around 1829.2, 1966.7, 1362.0 and 2174.5 MPa, for PLA, PLA-GNP, PETG and PETG-C_f_ materials, respectively. Their spectroscopic characterization revealed that graphene in PLA-GNP had good crystallinity, without significant level structural defects, and was in the form of nanoplatelets of 10–60 graphenic layers. Statistical filler distribution features were assessed by optical microscopy. Nozzle temperatures for the various filaments were established using DSC analysis of the raw materials, whereas the measured yield strength of 55 MPa served as an input for the tribometric measurements. Profilometry measurements of the as-printed samples provided a mean surface roughness of 37 μm, which was common for both geometric and biomimetic structures and fell within the range of printing resolution of 120 μm.

For the geometric cubic patterned surfaces, it was found that graphene nano-fillers reduced the friction coefficient of PLA by ca. 8%, from 0.2149 ± 0.030 to 0.1981 ± 0.025, whereas PETG exhibited even lower coefficient values of 0.0978 ± 0.035, the lowest in the study, indicating a high superlubric potential; the coefficient increased to 0.1191 ± 0.035 with carbon fibers oriented along the fused deposition direction. The corresponding behavior of biomimetic snakeskin patterns was characterized by considerable background noise and an inferior frictional response, with the relevant coefficient ranging between 0.20 and 0.27, due to the topologically and tribologically anisotropic surface, with preferential lubricity along the scale direction, rather than the circular movement of the tribometric counter body. Overall, the presence of graphene as a nano-filler in the starting materials appears to exhibit great potential for the reduction of friction between sliding surfaces, while PETG shows a tribological performance not only superior to PLA but, most importantly, compatible with expectations for superlubric performance.

## Figures and Tables

**Table 1:** Main mechanical properties of as-printed materials.

Material	Young’s Modulus [MPa]	Yield Strength [MPa]	Tensile Stress at Break [MPa]
PLA	1829.2 ± 44.5	47.03 ± 6.2	45.23 ± 6.8
PLA-GNP	1966.7 ± 48.3	49.60 ± 7.1	46.15 ± 7.0
PETG	1362.0 ± 27.5	46.97 ± 6.5	39.88 ± 5.9
PETG-C_f_	2174.5 ± 55.8	47.96 ± 8.3	41.75 ± 7.8

**Table 2:** Overview of measured ball-on-disk friction coefficients for all materials and patterns.

Material	Friction Coefficient [-]	
	Geometric Pattern	Biomimetic Pattern
PLA	0.2149 ± 0.030	0.2150 ± 0.093
PLA-GNP	0.1981 ± 0.025	0.2706 ± 0.093
PETG	0.0978 ± 0.035	0.0457 ± 0.070
PETG-C_f_	0.1191 ± 0.035	0.2053 ± 0.100

## Data Availability

Data are contained within the article.
